# Supplementary figures and images for: Direct evidence of megamammal-carnivore interaction decoded from bone marks in historical fossil collections from the Pampean region
Source: PeerJ. 2017 May 9;5:e3117. doi: 10.7717/peerj.3117 (PMC5426367; doi:10.7717/peerj.3117)

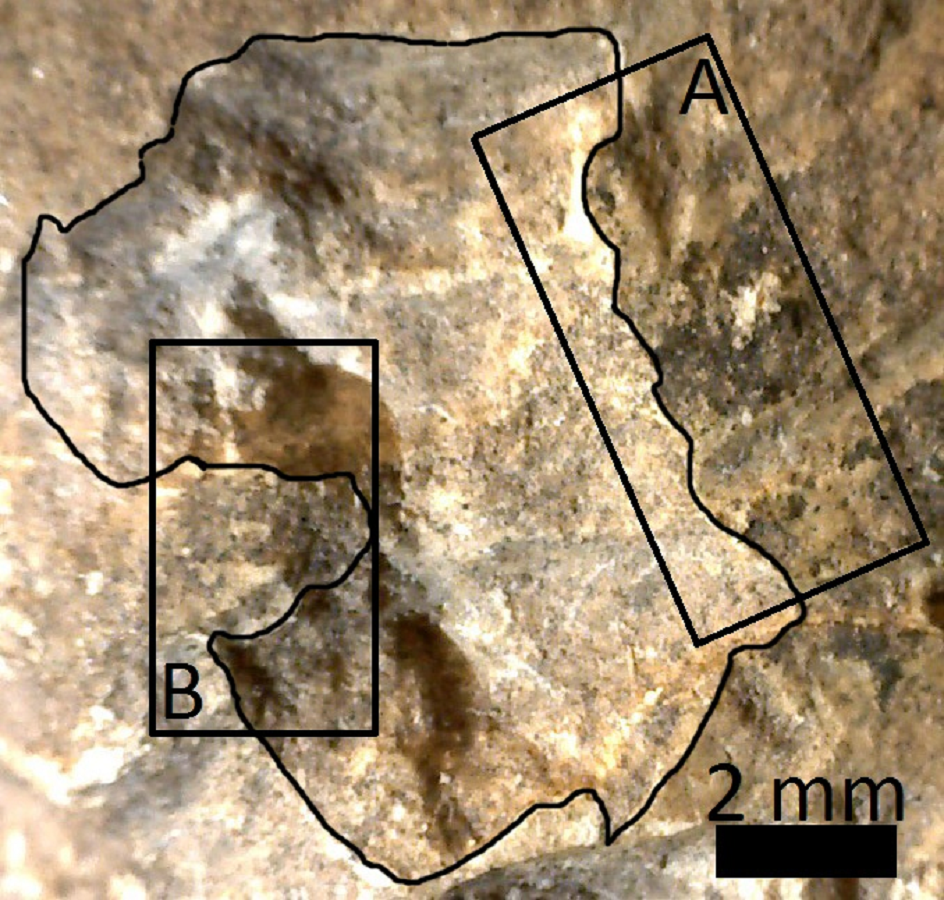

Supplement: Figure S1 — (A) Medial border where manganese spot abruptly ends. (B) Lateral border where the pit edge protrudes inwards. [file peerj-05-3117-s001.png]
