# Supplementary material for: Direct evidence of megamammal-carnivore interaction decoded from bone marks in historical fossil collections from the Pampean region
Source: PeerJ. 2017 May 9;5:e3117. doi: 10.7717/peerj.3117 (PMC5426367; doi:10.7717/peerj.3117)
Supplement: Table S1 [file peerj-05-3117-s002.docx]

| CARNIVORE MARKS | GENERAL CHARACTERISITC | CHARACTERISATION BY CARNIVORE GROUP | REFERENCES |
| --- | --- | --- | --- |
| PITTING AND/OR PUNCTURES | (i) Produced by the pressure of tooth on bone | URSIDS: Pitting will be planar, flat-bottomed, superficial and circular or square/rectangular. | Binford, 1981; Haynes, 1982, 1983; Lyman, 1994; Domínguez-Rodrigo and Piqueras, 2003; Pickering et al., 2004; Delaney-Rivera et al., 2009; Burke, 2013; Saladié et al., 2013; Arilla et al., 2014; Sala & Arsuaga, 2016 |
|  | (ii) Can leave a superficial imprint (pitting) or deeper mark (puncture) | FELIDS: These can inflict important teeth marks that have an “axe-edge” or elongated V-shape. |  |
|  | (iii) Depth depends on the amount of pressure exerted and whether this occurs on the softer cancellous bone of the epiphysis or on the harder part of the shaft | CANIDS: Tooth impressions tend to have a cone or truncated-cone shape. |  |
| SCRATCHES OR SCORES | (i) U-shaped | URSIDS: Characterised by short, wide, parallel groups or disordered and superimposed clusters of scratches with U-shape or, in some cases, quadrangular form. They can also leave elongated gouges. | Binford, 1981; Haynes, 1983; Lyman, 1994; Domínguez-Rodrigo and Piqueras, 2003; Pickering et al., 2004; Delaney-Rivera et al., 2009; Yravedra, Lagos & Bárcena, 2011; Burke, 2013; Saladié et al., 2013; Sala, Arsuaga & Haynes, 2014; Sala & Arsuaga, 2016 |
|  | (ii) Realised when teeth are dragged over a surface | FELIDS: Scoring will tend to be perpendicular to the long axis of the bone. |  |
|  | (iii) Can be superficial or present as gouges | CANIDS: These can leave numerous scores with different orientations. |  |
| FURROWING |  | URSIDS: These can crush, furrow, grind and leave crenulated edges. | Haynes, 1980, 1983; Binford, 1981; Lyman, 1994; Martin, 2008, 2016; Yravedra, Lagos & Bárcena, 2011; Burke, 2013; Saladié et al., 2013; Arilla et al., 2014; Sala, Arsuaga & Haynes, 2014; Domínguez-Rodrigo et al., 2015; Sala & Arsuaga, 2016 |
|  | (i) Cancellous bone extraction from the epiphyses | FELIDS: Some groups, such as jaguars, can furrow the epiphyses. |  |
|  | (ii) This action also can leave a crenulated edge, caused by the border of collapsed bone produced by the bite having an irregular edge | CANIDS: These have ample furrowing capacity |  |
| SPIRAL FRACTURES |  | URSIDS: Reduced bone breaking capacity. | Binford, 1981; Haynes, 1982; 1983; Capaldo & Blumenschine, 1994; Lyman, 1994; Yravedra, Lagos & Bárcena, 2011; Domínguez-Rodrigo et al., 2012; Saladié et al., 2013; Sala, Arsuaga & Haynes, 2014; Sala & Arsuaga, 2016 |
|  | (i) Fresh bone being broken due to pressure from the tooth leaving spiral borders | FELIDS: Reduced bone breaking capacity. |  |
|  | (ii) Sometimes this action leaves notches in the wall of the bone | CANIDS: These can crush and break epiphyses and diaphyses |  |
