# Supplementary material for: Direct evidence of megamammal-carnivore interaction decoded from bone marks in historical fossil collections from the Pampean region
Source: PeerJ. 2017 May 9;5:e3117. doi: 10.7717/peerj.3117 (PMC5426367; doi:10.7717/peerj.3117)
Supplement: Table S2 [file peerj-05-3117-s003.docx]

| De los Reyes et al., 2013 (Table 1) | | |
| --- | --- | --- |
| Specimen | Area mm^2^ |  |
| XEN 30-12 | 33.93 | 1.53058386 |
|  | 45.56 | 1.658583715 |
|  | 10.92 | 1.038222638 |
|  | 30.98 | 1.491081413 |
|  | 13.02 | 1.114610984 |
|  | 17.12 | 1.23350376 |
