# Supplementary material for: Direct evidence of megamammal-carnivore interaction decoded from bone marks in historical fossil collections from the Pampean region
Source: PeerJ. 2017 May 9;5:e3117. doi: 10.7717/peerj.3117 (PMC5426367; doi:10.7717/peerj.3117)
Supplement: Table S3 [file peerj-05-3117-s004.docx]

| Martin, 2016 |  |  |  |  |
| --- | --- | --- | --- | --- |
| Specimen | Length | Breadth | Area | Log Area |
| 94-VIII-10-1 | 41.63 | 30.36 | 1263.8868 | 3.10170818 |
|  | 23.37 | 21.86 | 510.8682 | 2.70830887 |
|  | 7.1 | 5.01 | 35.571 | 1.55109607 |
|  | 55.3 | 40.29 | 2228.037 | 3.3479224 |
|  | 10.61 | 7.46 | 79.1506 | 1.89845421 |
|  | 6.13 | 5.14 | 31.5082 | 1.49842359 |
|  | 15.09 | 4.4 | 66.396 | 1.82214192 |
| 94-VIII-10-96 | 17.56 | 13.43 | 235.8308 | 2.37260052 |
| 94-VIII-10-24 | 7.99 | 8.64 | 69.0336 | 1.83906052 |
|  | 5.17 | 4.99 | 25.7983 | 1.41159109 |
|  | 6.84 | 8.3 | 56.772 | 1.75413419 |
