# Supplementary material for: Direct evidence of megamammal-carnivore interaction decoded from bone marks in historical fossil collections from the Pampean region
Source: PeerJ. 2017 May 9;5:e3117. doi: 10.7717/peerj.3117 (PMC5426367; doi:10.7717/peerj.3117)
Supplement: Table S4 [file peerj-05-3117-s005.docx]

| Pickering et al., 2004 (Appendix A) | | |  |  |
| --- | --- | --- | --- | --- |
| Specimen | Length | Breadth | Area | Log Area |
| SWK 19683 | 3.2 | 2.7 | 8.64 | 0.936513742 |
|  | 2.4 | 1.3 | 3.12 | 0.494154594 |
| SWK 27684 | 5.3 | 3.3 | 17.49 | 1.242789809 |
|  | 4.5 | 3.4 | 15.3 | 1.184691431 |
|  | 3.6 | 2.2 | 7.92 | 0.898725182 |
|  | 2.5 | 2.1 | 5.25 | 0.720159303 |
|  | 3.1 | 1.8 | 5.58 | 0.746634199 |
| SWK 287324 | 4.2 | 3 | 12.6 | 1.100370545 |
|  | 2.3 | 1.5 | 3.45 | 0.537819095 |
| SWK 29283 | 3.2 | 2.5 | 8 | 0.903089987 |
|  | 1.9 | 1.8 | 3.42 | 0.534026106 |
|  | 1.6 | 1.4 | 2.24 | 0.350248018 |
|  | 2.6 | 1.7 | 4.42 | 0.645422269 |
|  | 3 | 1.8 | 5.4 | 0.73239376 |
| SWK 30555 | 6.8 | 4 | 27.2 | 1.434568904 |
| SWK 30628 | 2.3 | 1.9 | 4.37 | 0.640481437 |
|  | 2.2 | 1.9 | 4.18 | 0.621176282 |
| SWK 35153 | 3.4 | 2.4 | 8.16 | 0.911690159 |
|  | 3.3 | 3 | 9.9 | 0.995635195 |
| SWK 36073 | 3.8 | 3.2 | 12.16 | 1.084933575 |
| SWK 36361 | 4.9 | 4.5 | 22.05 | 1.343408594 |
| SWK 36675 | 4 | 2.8 | 11.2 | 1.049218023 |
|  | 4.1 | 4 | 16.4 | 1.214843848 |
|  | 4.1 | 2.6 | 10.66 | 1.027757205 |
