# Supplementary material for: Direct evidence of megamammal-carnivore interaction decoded from bone marks in historical fossil collections from the Pampean region
Source: PeerJ. 2017 May 9;5:e3117. doi: 10.7717/peerj.3117 (PMC5426367; doi:10.7717/peerj.3117)
Supplement: Table S5 [file peerj-05-3117-s006.docx]

|  | AREA OF PITTING/PUNCTURE (in mm) |  |  |  | LOG AREA |  |  |
| --- | --- | --- | --- | --- | --- | --- | --- |
| MCNV 64-492 | MNHN.F. PAM 119 | MNW 1908.XI.110 | MLP | MCNV 64-492 | MNHN.F. PAM 119 | MNW 1908.XI.110 | MLP |
| 12 | 48 | 51 | 64 | 1.079181246 | 1.681241237 | 1.707570176 | 1.806179974 |
| 15 | 49 |  | 18 | 1.176091259 | 1.69019608 |  | 1.255272505 |
| 54 | 30 |  | 7 | 1.73239376 | 1.477121255 |  | 0.84509804 |
| 20 | 9 |  | 26 | 1.301029996 | 0.954242509 |  | 1.414973348 |
|  |  |  | 4 |  |  |  | 0.602059991 |
