# Supplementary material for: Direct evidence of megamammal-carnivore interaction decoded from bone marks in historical fossil collections from the Pampean region
Source: PeerJ. 2017 May 9;5:e3117. doi: 10.7717/peerj.3117 (PMC5426367; doi:10.7717/peerj.3117)
Supplement: Table S6 [file peerj-05-3117-s007.docx]

| BOX | TAXONOMICAL DETERMINATION | ELEMENT | PITTING AND/OR PUNCTURES | SCRATCHES OR SCORES | SPIRAL FRACTURES | CRENULATED EDGES | TOTAL BONES WITH MARKS |
| --- | --- | --- | --- | --- | --- | --- | --- |
| 1 | Indeterminate | Diaphysis | 1 |  | 8 |  | 8 |
| 4 | Indeterminate | Diaphysis |  |  | 1 |  | 1 |
| 6 | Indeterminate | Diaphysis |  |  | 1 |  | 1 |
| 8 | Indeterminate | Diaphysis |  |  | 4 | 1 | 4 |
| 8 | Indeterminate | Diaphysis |  | 1 | 1 |  | 1 |
| 8 | Indeterminate | Diaphysis |  |  | 1 | 1 | 1 |
| 10 | Indeterminate | Diaphysis |  |  | 1 |  | 1 |
| 10 | Indeterminate | Diaphysis | 1 | 1 | 2 | 1 | 2 |
| 12 | Indeterminate | Diaphysis |  |  | 1 | 1 | 1 |
| 13 | Indeterminate | Diaphysis |  |  | 1 |  | 1 |
| 6 | Indeterminate | Diaphysis |  | 1 | 1 |  | 1 |
| 5 | Toxodontidae | Femur |  | 1 |  |  | 1 |
| 6 | Indeterminate | Indeterminate | 1 |  |  |  | 1 |
| 14 | Indeterminate | Indeterminate | 1 |  |  |  | 1 |
|  |  | TOTALS | 4 | 4 | 22 | 4 | 25 |
