# Supplementary material for: Direct evidence of megamammal-carnivore interaction decoded from bone marks in historical fossil collections from the Pampean region
Source: PeerJ. 2017 May 9;5:e3117. doi: 10.7717/peerj.3117 (PMC5426367; doi:10.7717/peerj.3117)
Supplement: Supplemental Information 1 [file peerj-05-3117-s008.docx]

**Data S1. Description of carnivore marks**

(i) MCNV 64-492: On the right tibia of cf. Scelidotheriinae gen. the marks are concentrated on the distal epiphysis and medial face and, to a lesser degree, on the proximal epiphysis (Fig. 4). The distal epiphysis has a different groups of marks (Fig. 4A, 4B). Near the medial edge of the articular face is where most damage is observed. Here, four superficial pits are positioned linearly and surrounded by scratches (Fig. 4A). Posteriorly-anteriorly oriented, the first two pits are slightly smaller with a cuspid shape, while the other two are bigger. The pit 9 x 6 mm is almost double the size of the others. This mark was affected by post-depositional agents that probably resulted in this size increase. The manganese spot located next to the lateral side of the pit ends abruptly at the border and does not continue inside (Figure S1A). Additionally, almost in the middle, the medial border protrudes inwards, into the pit, as if the bone originally continued, separating this pit into two (Figure S1B). In consequence, it seems likely that this pit began as two, and that post-depositional events debilitated the bony separation between them. In this sense the edges of bone pits can be more affected by post-depositional conditions, as their surroundings become more susceptible to flaking and localised damage (Delaney-Rivera et al. 2009). On the lateral side of the distal articular face (Fig. 4A), a larger transverse score was detected. Parallel U-shaped scores are located over the metadiaphysis that continue beyond the rim with the four pits. One group of scores depart from the furrowing towards the articular side, while another starts from the articular side and runs towards the furrowing (Fig. 4B). They run parallel to the long axis of the bone and surround significant furrowing. This pattern implies that the *tibia caudalis* and *flexor digitorium longus* muscles were removed (Fig. 4C). Another significant furrow is present on the medial face of the proximal epiphysis (Fig. 4E); this has extracted part of the inner condyle. A crenulated rim surrounds this furrow, and there are parallel, V-shaped tooth marks over the posterior face (Fig. 4C and Fig. 4E). There is one group of five marks on the distal part and two on the proximal part, oriented posteriorly-medially. Three thick quadrangular-shaped grooves were detected on the medial face of the diaphysis (Fig. 4D). One runs along the entire face; the other two are smaller and more superficial. They start at the border of the anterior face and run up to the medial face (see Table 2 for measurements).

(ii) MNHN.F.PAM 119: The marks detected that are attributable to carnivores are on the distal epiphysis of the left humerus of *Glossotherium robustum* (Fig. 5). They are distributed on the articular face, over the condyle and trochlear regions (Fig. 5A). Near the medial side of the trochlear region, there are several V-shape punctures, surrounded by scratches (Fig. 5B). Part of the trochlea has disappeared and there are crenulated edges as a consequence of the furrowing. On the condyle, at least seven scores were detected (Fig. 5C), four of which are parallel. Superficial scratches were also observed. In the border of this region, over the lateral side, are two wide grooves (Fig. 5D) (see Table 2 for measurements).

(iii) 1908. XI.110: On the left humerus of *Glossotherium robustum* housed at the MNW, there is a corrugated fracture over the lateral face of the condyle that encompasses both anterior and posterior faces (Fig. 6A and Fig.6B). The epicondyle has been destroyed and the border has a crenulated edge. The collapsed bone is covered with sediment and the rim of the fracture is the same colour as the rest of the specimen: thus the fracture must have occurred prior to burial. Although the furrowing and crenulated edge is weak evidence of carnivore intervention (Domínguez-Rodrigo et al., 2015), the deltoid crest of the posterior face also has a possible puncture with sediment inside (Fig. 6B). Additionally, in the posterior view, the fractured border is flaked resulting from pressure exerted on it (Fig.6C and Fig.6D). The regularity of the fracturing on both the anterior and posterior faces supports the proposal that the marks on this bone could have resulted from the action of carnivores (see Table 2 for measurements).

(iv) From the megamammal bones in the MLP assemblage, a condyle of a distal femur of Toxodontidae was identified, with eight elongated, short, long and U-shaped scratches (Fig. 7). In addition, 22 bone shafts from smaller unidentified mammals display spiral fractures. Some of these also present scratches, crenulated edges or light pitting (Figs. 8 and 9). Semi-circular notches were also identified. Two indeterminate bones have bigger punctures (Fig. 10). Spiral fractures can be confused with human intervention or can occur naturally (Binford, 1981; Lyman, 1994). Nevertheless, the presence of other typical carnivore damage such as scratches and perforations, enables us to consider them as being produced by carnivore activity (see Table 2 for measurements and detail of marks in Table S6).

**Data S2. Description of Pampean carnivores**

(i) The ursid *Arctotherium angustidens* evolved during the Ensenadan Stage/Age. This large ‘short-faced’ bear was a member of the megafauna and recent estimations of its body mass indicate that the animal weighed more than a tonne (Soibelzon et al., 2014). Recent morphometric studies also indicate that this bear probably had an omnivorous diet supplemented by meat or carrion, as dental pathologies detected in some individuals of *Arctotherium* probably resulted from chewing bones (Figueirido & Soibelzon, 2010). Moreover, Soibelzon et al. (2014) have found biomechanical and isotopic evidence of *A. angustidens* having an omnivorous diet but with scavenging abilities. Other smaller bears that appeared later in South America, including *Arctotherium vetustum*, *Arctotherium bonariense* and *Arctotherium tarijense*, had a more plant-based diet (Figueirido & Soibelzon, 2010).

(ii) Three felids were also present in these ecosystems. The dirk-toothed sabre cat *Smilodon populator* was the top predator in this region: its estimated body mass has been calculated as being between 220-360 kg, but it could have reached up to 400 kg (Christiansen & Harris, 2006). This sabre-toothed cat may even have been capable of hunting juvenile *Megatherium americanum* (Tardigrada, Megatheriidae), with a body mass of adult individuals ranging between 4.000 and 6.000 kg (Prevosti & Vizcaíno, 2006; Bocherens et al., 2016). However, the large sabre-like canines that it used to attack to the throat of its prey (Antón et al., 2004) precluded *Smilodon* from breaking or consuming bones regularly, although they could have inflicted important bone damage during hunting and/or soft-tissues consumption (Van Valkeburgh & Hertel, 1993; Marean & Ehrhardt 1995; Binder & Van Valkenburgh, 2010). The other two hypercarnivorous felids were *Puma concolor,* with an estimated body mass of 47-50 kg (Christiansen & Harris, 2006; Prevosti & Vizcaíno, 2006), and *Panthera onca,* weighing *ca.* 120 kg (Prevosti & Vizcaíno, 2006). Although these species would have fed on prey of *ca*. 600 kg; occasionally theses preyed on juvenile megamammals (Prevosti &Vizcaíno, 2006). The puma could have inflicted substantial mark on bone but would not usually have consumed it (Muñoz et al., 2008; Kaufmann et al. 2016). In contrast, *Panthera onca* was potentially able to break and consume bone (Martín, 2008; Domínguez-Rodrigo et al., 2015).

(iii) Hypercarnivorous canids were also present in these ecosystems at the same time. They could have cooperated in order to hunt large mammals and juvenile megamammals, and they would also have had the ability to scavenge (Prevosti & Palmqvist, 2001; Prevosti, Zurita & Carlini, 2005; Prevosti & Schubert, 2013). This may have been the case for *Theriodictis platensis*, weighing *ca*. 37 kg, which evolved during the Ensenadan Stage/Age. It could have preyed upon animals of around 600 kg, animals of extreme age classes (i.e., very old or juvenile individuals), or diseased members of the megafauna (Prevosti & Palmqvist, 2001). During the Pleistocene, there were various species of *Protocyon*, weighing between 20 and 25 kg. These could have hunted middle-sized mammals, scavenged carcasses of megamammals, and may even have competed with *Smilodon populator* (Prevosti, Zurita & Carlini, 2005; Prevosti & Schubert, 2013; Bocherens et al., 2016). *Canis nehringui,* weighing *ca*. 32 kg, was present during the late Pleistocene-early Holocene and although it would have generally fed on medium-sized mammals, pack-hunting of bigger species may have been possible (Prevosti & Vizcaíno, 2006). *Dusicyon avus*, weighing *ca*. 14 kg, would have specialised in smaller species, but consumption of larger mammals cannot be ruled out (Prevosti & Vizcaíno, 2006).
