# Supplementary material for: Direct evidence of megamammal-carnivore interaction decoded from bone marks in historical fossil collections from the Pampean region
Source: PeerJ. 2017 May 9;5:e3117. doi: 10.7717/peerj.3117 (PMC5426367; doi:10.7717/peerj.3117)
Supplement: Data S1 [file peerj-05-3117-s009.docx]

**Data S1. Description of carnivore marks**

(i) MCNV 64-492: On the right tibia of cf. Scelidotheriinae gen. the marks are concentrated on the distal epiphysis and medial face and, to a lesser degree, on the proximal epiphysis (Fig. 4). The distal epiphysis has a different groups of marks (Fig. 4A, 4B). Near the medial edge of the articular face is where most damage is observed. Here, four superficial pits are positioned linearly and surrounded by scratches (Fig. 4A). Posteriorly-anteriorly oriented, the first two pits are slightly smaller with a cuspid shape, while the other two are bigger. The pit 9 x 6 mm is almost double the size of the others. This mark was affected by post-depositional agents that probably resulted in this size increase. The manganese spot located next to the lateral side of the pit ends abruptly at the border and does not continue inside (Figure S1A). Additionally, almost in the middle, the medial border protrudes inwards, into the pit, as if the bone originally continued, separating this pit into two (Figure S1B). In consequence, it seems likely that this pit began as two, and that post-depositional events debilitated the bony separation between them. In this sense the edges of bone pits can be more affected by post-depositional conditions, as their surroundings become more susceptible to flaking and localised damage (Delaney-Rivera et al. 2009). On the lateral side of the distal articular face (Fig. 4A), a larger transverse score was detected. Parallel U-shaped scores are located over the metadiaphysis that continue beyond the rim with the four pits. One group of scores depart from the furrowing towards the articular side, while another starts from the articular side and runs towards the furrowing (Fig. 4B). They run parallel to the long axis of the bone and surround significant furrowing. This pattern implies that the *tibia caudalis* and *flexor digitorium longus* muscles were removed (Fig. 4C). Another significant furrow is present on the medial face of the proximal epiphysis (Fig. 4E); this has extracted part of the inner condyle. A crenulated rim surrounds this furrow, and there are parallel, V-shaped tooth marks over the posterior face (Fig. 4C and Fig. 4E). There is one group of five marks on the distal part and two on the proximal part, oriented posteriorly-medially. Three thick quadrangular-shaped grooves were detected on the medial face of the diaphysis (Fig. 4D). One runs along the entire face; the other two are smaller and more superficial. They start at the border of the anterior face and run up to the medial face (see Table 2 for measurements).

(ii) MNHN.F.PAM 119: The marks detected that are attributable to carnivores are on the distal epiphysis of the left humerus of *Glossotherium robustum* (Fig. 5). They are distributed on the articular face, over the condyle and trochlear regions (Fig. 5A). Near the medial side of the trochlear region, there are several V-shape punctures, surrounded by scratches (Fig. 5B). Part of the trochlea has disappeared and there are crenulated edges as a consequence of the furrowing. On the condyle, at least seven scores were detected (Fig. 5C), four of which are parallel. Superficial scratches were also observed. In the border of this region, over the lateral side, are two wide grooves (Fig. 5D) (see Table 2 for measurements).

(iii) 1908. XI.110: On the left humerus of *Glossotherium robustum* housed at the MNW, there is a corrugated fracture over the lateral face of the condyle that encompasses both anterior and posterior faces (Fig. 6A and Fig.6B). The epicondyle has been destroyed and the border has a crenulated edge. The collapsed bone is covered with sediment and the rim of the fracture is the same colour as the rest of the specimen: thus the fracture must have occurred prior to burial. Although the furrowing and crenulated edge is weak evidence of carnivore intervention (Domínguez-Rodrigo et al., 2015), the deltoid crest of the posterior face also has a possible puncture with sediment inside (Fig. 6B). Additionally, in the posterior view, the fractured border is flaked resulting from pressure exerted on it (Fig.6C and Fig.6D). The regularity of the fracturing on both the anterior and posterior faces supports the proposal that the marks on this bone could have resulted from the action of carnivores (see Table 2 for measurements).

(iv) From the megamammal bones in the MLP assemblage, a condyle of a distal femur of Toxodontidae was identified, with eight elongated, short, long and U-shaped scratches (Fig. 7). In addition, 22 bone shafts from smaller unidentified mammals display spiral fractures. Some of these also present scratches, crenulated edges or light pitting (Figs. 8 and 9). Semi-circular notches were also identified. Two indeterminate bones have bigger punctures (Fig. 10). Spiral fractures can be confused with human intervention or can occur naturally (Binford, 1981; Lyman, 1994). Nevertheless, the presence of other typical carnivore damage such as scratches and perforations, enables us to consider them as being produced by carnivore activity (see Table 2 for measurements and detail of marks in Table S6).
