# Supplementary material for: Direct evidence of megamammal-carnivore interaction decoded from bone marks in historical fossil collections from the Pampean region
Source: PeerJ. 2017 May 9;5:e3117. doi: 10.7717/peerj.3117 (PMC5426367; doi:10.7717/peerj.3117)
Supplement: Data S2 [file peerj-05-3117-s010.docx]

**Data S2. Description of Pampean carnivores**

(i) The ursid *Arctotherium angustidens* evolved during the Ensenadan Stage/Age. This large ‘short-faced’ bear was a member of the megafauna and recent estimations of its body mass indicate that the animal weighed more than a tonne (Soibelzon et al., 2014). Recent morphometric studies also indicate that this bear probably had an omnivorous diet supplemented by meat or carrion, as dental pathologies detected in some individuals of *Arctotherium* probably resulted from chewing bones (Figueirido & Soibelzon, 2010). Moreover, Soibelzon et al. (2014) have found biomechanical and isotopic evidence of *A. angustidens* having an omnivorous diet but with scavenging abilities. Other smaller bears that appeared later in South America, including *Arctotherium vetustum*, *Arctotherium bonariense* and *Arctotherium tarijense*, had a more plant-based diet (Figueirido & Soibelzon, 2010).

(ii) Three felids were also present in these ecosystems. The dirk-toothed sabre cat *Smilodon populator* was the top predator in this region: its estimated body mass has been calculated as being between 220-360 kg, but it could have reached up to 400 kg (Christiansen & Harris, 2006). This sabre-toothed cat may even have been capable of hunting juvenile *Megatherium americanum* (Tardigrada, Megatheriidae), with a body mass of adult individuals ranging between 4.000 and 6.000 kg (Prevosti & Vizcaíno, 2006; Bocherens et al., 2016). However, the large sabre-like canines that it used to attack to the throat of its prey (Antón et al., 2004) precluded *Smilodon* from breaking or consuming bones regularly, although they could have inflicted important bone damage during hunting and/or soft-tissues consumption (Van Valkeburgh & Hertel, 1993; Marean & Ehrhardt 1995; Binder & Van Valkenburgh, 2010). The other two hypercarnivorous felids were *Puma concolor,* with an estimated body mass of 47-50 kg (Christiansen & Harris, 2006; Prevosti & Vizcaíno, 2006), and *Panthera onca,* weighing *ca.* 120 kg (Prevosti & Vizcaíno, 2006). Although these species would have fed on prey of *ca*. 600 kg; occasionally theses preyed on juvenile megamammals (Prevosti &Vizcaíno, 2006). The puma could have inflicted substantial mark on bone but would not usually have consumed it (Muñoz et al., 2008; Kaufmann et al. 2016). In contrast, *Panthera onca* was potentially able to break and consume bone (Martín, 2008; Domínguez-Rodrigo et al., 2015).

(iii) Hypercarnivorous canids were also present in these ecosystems at the same time. They could have cooperated in order to hunt large mammals and juvenile megamammals, and they would also have had the ability to scavenge (Prevosti & Palmqvist, 2001; Prevosti, Zurita & Carlini, 2005; Prevosti & Schubert, 2013). This may have been the case for *Theriodictis platensis*, weighing *ca*. 37 kg, which evolved during the Ensenadan Stage/Age. It could have preyed upon animals of around 600 kg, animals of extreme age classes (i.e., very old or juvenile individuals), or diseased members of the megafauna (Prevosti & Palmqvist, 2001). During the Pleistocene, there were various species of *Protocyon*, weighing between 20 and 25 kg. These could have hunted middle-sized mammals, scavenged carcasses of megamammals, and may even have competed with *Smilodon populator* (Prevosti, Zurita & Carlini, 2005; Prevosti & Schubert, 2013; Bocherens et al., 2016). *Canis nehringui,* weighing *ca*. 32 kg, was present during the late Pleistocene-early Holocene and although it would have generally fed on medium-sized mammals, pack-hunting of bigger species may have been possible (Prevosti & Vizcaíno, 2006). *Dusicyon avus*, weighing *ca*. 14 kg, would have specialised in smaller species, but consumption of larger mammals cannot be ruled out (Prevosti & Vizcaíno, 2006).
